# Diabetic kidney disease in patients with type 2 diabetes mellitus: a cross-sectional study

**DOI:** 10.1186/s12882-021-02429-4

**Published:** 2021-06-16

**Authors:** Randa I. Farah, Mohammed Q. Al-Sabbagh, Munther S. Momani, Asma Albtoosh, Majd Arabiat, Ahmad M. Abdulraheem, Husam Aljabiri, Mohammad Abufaraj

**Affiliations:** 1 Department of Internal Medicine, School of Medicine, University of Jordan, Amman, Jordan; 2 School of medicine, University of Jordan, Amman, Jordan; 3 Division of Urology, Department of Special Surgery, Jordan University Hospital, The University of Jordan, Amman, Jordan; 4 Department of Urology, Medical University of Vienna, Vienna, Austria

**Keywords:** DKD, Albuminuria, Type 2 DM, Metformin, Dyslipidemia

## Abstract

**Aim:**

Diabetic kidney disease (DKD) is a major long-term complication of diabetes mellitus (DM). Given the paucity of data on DKD in Jordan, we aimed to evaluate the prevalence, characteristics and correlates of DKD in Jordanian patients with type 2 DM.

**Methods:**

This cross-sectional study included 1398 adult patients with type 2 DM who sought medical advice in the endocrinology clinic between March and September 2019. Demographic, clinical and laboratory data were reviewed. DKD was defined as reduced eGFR, and/or albuminuria. Three regression models were constructed to identify factors associated with CKD stages, albuminuria and DKD.

**Results:**

Overall, 701 (50.14%) patients had DKD, with a median age of 59.71 ± 11.36  years. Older age, high triglycerides, and low high-density lipoprotein were associated with DKD (multivariable odds ratio [OR]: 1.02, 95% confidence interval [CI]: 1.01–1.03, *p* < 0.01; OR: 1.1, 95% CI: 1.01–1.2; and OR: 0.98, 95% CI: 0.97–0.99, *p* < 0.01 respectively). Metformin and renin-angiotensin system blockers were negatively associated with albuminuria and chronic kidney disease stages (*p* < 0.01).

**Conclusion:**

Our study demonstrated that approximately one half of patients with type 2 DM had DKD. Further studies are necessary to understand this high prevalence and the underlying factors. Future research are needed to assess implementing targeted community-based intervention.

## Introduction

Worldwide, diabetes mellitus (DM) is a growing healthcare challenge and imposes a heavy burden on public health [1]. DM type 2 accounts for more than 90% of diabetes cases, and there is a rising number of people diagnosed with diabetes type 2 [1] with more rapid increase in low- and middle-income countries than in high-income countries [2].

Diabetic kidney disease (DKD) is a major long-term complication of DM type 2 and is the leading cause of chronic kidney disease (CKD) and end-stage kidney disease (ESKD) worldwide [3]. Although renal biopsy is the gold standard to diagnose diabetic nephropathy, the majority of diabetic patients do not undergo kidney biopsy, as they are presumed to have diabetic kidney disease based upon clinical history and laboratory evaluation and because of invasive nature of kidney biopsy [4]. Furthermore, an increasing number of DM type 2 patients present with DKD [5]. The incidence and rate of DKD are less clear in DM type 2 than in type 1, mainly due to the highly variable age of onset and difficulty in defining the exact time of onset and associated comorbidities [6].

There are marked racial differences in the epidemiology of DKD, which could be partially explained by the differences in health care services and environmental factors such as dietary intake and smoking [6]. Studying the prevalence and factors associated with DKD in patients with DM type 2 is crucial to estimate the disease’s burden and modifiable risk factors and to set research priorities. The urbanization, rapid change in the lifestyle and the dramatic increase in type 2 diabetes prevalence in Jordan necessitate evaluating the consequences of this disease, such as DKD [2].

Although the prevalence of DM type 2 in Jordan has been shown to be on the rise [2], the prevalence of DKD risk factors has not been well elucidated. Moreover, a focused study on DKD degree/severity among those with DM type 2 in Jordan has not been reported. Therefore, we aim to evaluate the prevalence, characteristics, and risk factors of DKD in patients with DM type 2 in Jordan. These findings will help define priorities and establish health policies on the national level and aid in patient counseling and treatment planning.

## Materials and methods

### Study population

This cross-sectional study aimed to assess the prevalence of DKD among patients aged >18 years who were diagnosed with type 2 diabetes and sought medical advice at the University of Jordan Hospital, Endocrinology clinic, between March and September 2019. We excluded patients with missing data (those that did not have at least two readings of serum creatinine or those that did not have at least two readings of urine albumin protein within 6 months of the assigned clinic visit), ESKD on dialysis, and other possible causes of CKD or proteinuria. Informed consent was obtained from all participants. This study was approved by the University of Jordan Institutional Review Board and followed the institutional and/or national research committee’s ethical standards and the principles of the World Medical Association Declaration of Helsinki.

### Data collection and evaluations

Patients diagnosed with DM type 2 were identified according to patients’ medical records and verified during their clinic visits by an assigned research assistant. The following data were collected via face-to-face interviews and referral to medical charts by a trained research assistant: medical history including hypertension, myocardial infarction, coronary artery disease, heart failure, stroke, and the onset of diabetes; current diabetes status with complications such as retinopathy, peripheral neuropathy, and cardiovascular disease as reported during the interview; data on glycemic control, i.e., latest values of fasting blood glucose and hemoglobin A1c (HbA1c); and smoking status. Current medication usage, such as antidiabetics, antihypertensives, lipid-lowering agents, and other medications, taken by the patients was also reviewed. A trained research assistant performed the following measurements in the office: blood pressure (mean of two consecutive measurements of systolic and diastolic blood pressure in the sitting position, 3–4 min apart) and we considered abnormal blood pressure reading >140 mmHg systolic and >90 mmHg diastolic, height, weight, and waist circumference. We also collected data on HbA1c, fasting blood sugar, fasting lipid profile (if available) as we considered abnormal results as following: High density lipoprotein (HDL <38 mg/dl in men and <50 mg/dl in women) and high triglyceride >160 mg/dl and Low density lipoprotein (LDL) >100 mg/dl, and serum albumin levels up to 3–6 months from the clinic visit using the latest blood test results when available. We assessed the kidney function using serum creatinine and urinary albumin excretion measurements on two occasions at least 3 months apart. We excluded patients who did not have 2 readings of any of the kidney function assessment within 6 months of the assigned clinic visit.

The estimated glomerular filtration rate (eGFR) was determined for each patient using a standardized serum creatinine level and the Chronic Kidney Disease Epidemiology Collaboration formula. Increased urinary albumin excretion was diagnosed as 1) microalbuminuria, if the urinary albumin to creatinine ratio (ACR) was ≥30 and ≤300 mg/g or the protein to creatinine ratio (PCR) was >0.150 and <0.500 g/g or 2) macroalbuminuria, if the ACR was ≥300 mg/g or the PCR was >0.500 g/g, and 3) albuminuria indicated patients with either micro- or macroalbuminuria [7, 8]. Urine samples that were positive for leucocytes and nitrites, which are indicative of significant urinary tract infection, and erythrocyte or Hb levels of ≥5 counts/μL, which is indicative of significant hematuria (false positives), were excluded.

DKD was defined as a reduced eGFR, <60 mL/min/1.73 m^2^, and/or increased urinary albumin excretion, ≥30 mg/g creatinine that persisted for ≥3 months in the presence of longstanding diabetes and exclusion of other causes of CKD.

### Statistical analysis

All analyses were performed using STATA (Stata Statistical Software: Release 16. College Station, TX, StataCorp LLC). Categorical variables are presented as percentages, while continuous variables are presented as the mean ± standard deviation.

Differences in sociodemographic characteristics, past medical history, comorbidities, and medications among patients with or without evidence of DKD were assessed using the chi-square test for categorical variables and Student’s t-test continuous variables. Three regression models were constructed: (i) to identify variables associated with CKD stages, a multistage ordinal logistic regression analysis was used, (ii) to assess variables associated with the degree of albuminuria, a multistage ordinal logistic regression analysis was used, and (iii) finally, a binary logistic regression was used to assess the variables associated with an increased risk of DKD. The confidence interval was set at 95%, and *p*-values of ≤0.05 were considered to indicate statistical significance.

## Results

Among 1652 patients, 1398 patients met our inclusion criteria. The median age was 59.71 ± 11.36 years, there were 573 men (40.99%) and 825 women (59.01%), and the median duration of DM type 2 was 10.42 ± 7.99 years. Further, 970 (69.38%) patients in our study population were hypertensive with a mean systolic blood pressure of 135.53 ± 21.37 mmHg, and the mean diastolic blood pressure was 77.72 ± 13.61 mmHg. A body mass index (BMI) of >30 was identified in 66% of the population. The mean HbA1c level was 7.71% ± 1.60%. Retinopathy was documented in 477 patients (34.12%). These baseline characteristics of our study population are summarized in Table 1.

**Table 1:** Patient’s characteristics according to diabetic kidney disease status

Variable	Category	n (%)	DKD n (%)	Non-DKD n (%)	*p*-value
		1398 (100)	701 (50.14)	697 (49.86)	
Age (Years)	<60	646 (46.21)	289 (41.23)	357 (51.22)	<0.01*
	≥60	752 (52.79)	412 (58.77)	340 (48.78)	
Sex	Male	572 (41)	287 (41)	286 (41)	0.97
	Female	825 (59)	414 (59)	411 (59)	
Duration of DM	<5 years	393 (28.1)	162 (23.1)	231 (33.1)	<0.01*
	5–10 years	304 (21.8)	142 (20.3)	162 (23.23)	
	>10 years	701 (50.1)	397 (56.6)	304 (43.6)	
HbA1c (%)	<7.0	547 (39.1)	248 (35.4)	299 (42.9)	<0.01*
	≥7.0	851 (60.9)	453 (64.6)	398 (57.1)	
Antidiabetic medications	Insulin	691 (49.5)	389 (55.6)	302 (43.4)	<0.01*
	Metformin	1074 (76.9)	475 (67.9)	500 (86)	<0.01*
	Other anti-diabetics	624 (44.7)	308 (44)	316 (45.4)	0.6
Using RAAS blockers		737 (52.8)	388 (55.4)	349 (50.1)	<0.05*
Waist circumference (cm)		109.3 ± 14.6*	110.2 ± 14.6*	108.4 ± 14.6*	0.02*
BMI	<25	121 (8.7)	58 (8.3)	63 (9)	0.87
	25–29	353 (25.3)	176 (25.2)	177 (25.5)	
	>30	920 (66)	464 (66.5)	456 (65.5)	
Smoking	Never smoker	986 (70.5)	488 (69.6)	498 (71.5)	0.18
	Current smoker	175 (12.5)	99 (14.1)	76 (11)	
	Ex-smoker	237 (17)	114 (16.3)	123 (17.5)	
Medical history	Hypertension	970 (69.4)	540 (77)	430 (61.7)	<0.01*
Medical history	Myocardial infarction	154 (11)	91 (13)	63 (9)	0.02*
	Congestive heart failure	46 (3.3)	26 (3.7)	20 (2.9)	0.38
	Coronary artery disease	155 (11.1)	92 (13.1)	63 (9)	0.02*
	Stroke	97 (6.9)	64 (9.1)	33 (4.7)	0.01*
	Neuropathy	596 (42.66)	330 (47.1)	266 (38.2)	0.01*
	Retinopathy	477 (34.1)	289 (41.2)	188 (27)	<0.01*

*Mean ± standard deviation

*DKD* diabetic kidney disease, *DM* diabetes mellitus, RAAS blockers, *BMI* body mass index

*Statistically significant

We found that 701 (50.14%) patients had DKD, 625 (44.7%) had albuminuria, and 268 (19.17%) had CKD with an eGFR of <60 mL/min/1.73 m^2^.

**Fig. 1:**
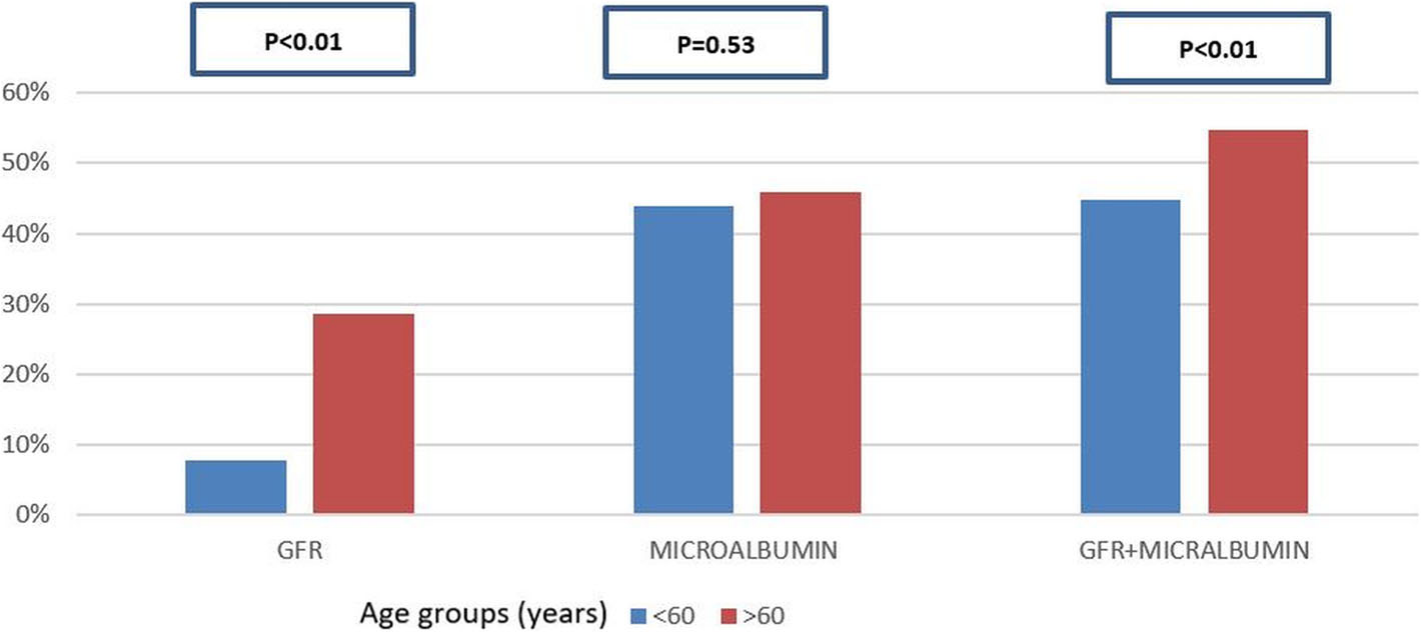
Proportion of patients with eGFR <60 mL/min/1.73 m^2^ or albuminuria, by age groups

DKD and a reduced eGFR were more prevalent among patients aged >60 years (*p* < 0.001) (Fig. 1). Such an association was not detected for microalbuminuria or albuminuria (*p* = 0.53). We also found that patients with DKD were more likely to have had used insulin, metformin, and renin-angiotensin system (RAAS) blockers (*p* < 0.01) than were those without DKD (*p* < 0.05) (Table 1).

On univariate analysis comparing patients with DKD to those without DKD, we found age >60 years, longer duration of diabetes, higher HbA1c of >7%, and hypertension were correlated to DKD (*p* < 0.0001). DKD was also associated retinopathy and neuropathy (*p* < 0.01).

Table 2 shows the multivariable analysis assessing factors associated with diabetic kidney disease. Age older than 60 year was associated with DKD (multivariable odds ratio [OR]: 1.02, 95% confidence interval [CI]: 1.01–1.03, *p* < 0.01). Retinopathy, neuropathy, high triglyceride levels, and low high-density lipoprotein (HDL) levels were also correlated with DKD (OR: 1.27, 95% CI: 1.01–1.6, *p* < 0.05; OR: 1.3, 95% CI: 1.01–1.7, *p* < 0.05; OR: 1.1, 95% CI: 1.01–1.2, *p* < 0.01; and OR: 0.98, 95% CI: 0.97–0.99, *p* < 0.01; respectively). Metformin was negatively correlated with DKD (OR: 0.42, 95% CI: 0.31–0.55, *p* < 0.01). The use of insulin, beta blockers, calcium channel blockers, and diuretics was also significantly associated with DKD (OR: 1.3, 95% CI: 1.1–1.7, *p* = 0.01; OR: 1.3, 95% CI: 1.01–1.7, *p* = 0.02; OR: 1.4, 95% CI: 1.1–1.9, *p* < 0.01; and OR: 1.4, 95% CI: 1.1–1.8, *p* = 0.01; respectively) (Table 2).

**Table 2:** Multivariate binary logistic regression analyses for factors associated with diabetic kidney disease

Variable	Subgroups	OR	95% CI	*p* value
**Sex**	Female (Ref)			
	Male	0.9	0.7–1.12	0.6
**Age (Years)**		1.02	1.01–1.03	0.02*
**Duration of DM (Years)**		1.1	0.99–1.03	0.42
**Medical history**				
**Smoking**		1.12	0.95–1.3	0.17
**Hypertension**		1.4	1.1–1.8	0.03*
**CAD**		0.9	0.6–1.3	0.44
**Stroke**		1.4	0.9–2.2	0.16
	Neuropathy	1.24	1.01–1.6	<0.05*
	Retinopathy	1.3	1.01–1.7	<0.05*
**Medications**	Insulin	1.38	1.1–1.7	0.01*
	Metformin	0.42	0.33–0.55	<0.01*
	ACEIs/ARBs	0.8	0.6–1.1	0.1
	Beta blockers	1.3	1.01–1.7	0.02*
	Diuretics	1.4	1.1–1.8	0.01*
	CCB	1.4	1.1–1.9	<0.01*
**Triglyceride’s level (mg/dl)**		1.1	1.01–1.2	<0.01*
**HDL (mg/dl)**		0.98	0.97–0.99	<0.01*

*OR* odds ratio, *CI* confidence interval, *DM* diabetes mellitus, *CAD* coronary artery disease, *ACEI* angiotensin converting enzyme inhibitor, *ARB* angiotensin receptor blocker, *CCB* calcium channel blocker, *HDL* high-density lipoprotein

*Statically significant

Advanced CKD was significantly associated with older age and diabetes duration (β coefficient: 0.06 and 0.03, *p* < 0.01 and <0.04, respectively). It was also strongly associated with the use of beta-blockers, diuretics, and calcium channel blockers (β coefficient: 0.35, 0.6, and 0.7, respectively; *p* < 0.01 for all three factors). We observed that RAAS blocker and metformin use were negatively associated with advanced CKD stage (β coefficient: 0.31 and −1.34, respectively; *p* < 0.01 for both factors) (Table 3).

**Table 3:** Multivariable ordinal logistic regression analyses evaluating the correlates of chronic kidney disease and its stage

Variable	Subgroups	Beta coefficient	95% CI	*p* value
**Sex**	Female (Ref)			
	Male	−0.2	−0.41–0.1	0.24
**Age (Years) (continuous)**		0.06	0.05–0.07	<0.01*
**Duration of DM (Y) (continuous)**		0.02	0.01–0.03	0.04*
**Medical history**	Smoking	−0.5	− 0.21–0.12	0.6
	Hypertension	0.23	−0.07–0.54	0.13
	CAD	0.12	−0.23–0.73	0.5
	Stroke	0.32	− 0.1–0.7	0.13
	Neuropathy	−0.1	−0.33–0.14	0.4
	Retinopathy	0.27	0.02–0.5	0.04*
**Medications**	Insulin	0.06	−0.2–0.31	0.65
	Metformin	−1.34	−1.6–-1.1	<0.01*
	ACEIs/ARBs	−0.31	− 0.56–-0.10	<0.01*
	Beta blockers	0.35	0.1–0.6	<0.01*
	Diuretics	0.6	0.33–0.85	<0.01*
	CCB	0.7	0.44–1	<0.01*
**Triglyceride level (mg/dl)**		0.01	0.0001–0.02	<0.01*

*CI* confidence interval, *DM* diabetes mellitus, *CAD* coronary artery disease, *ACEI* angiotensin converting enzyme inhibitor, *ARB* angiotensin receptor blocker, *CCB* calcium channel blocker

*Statically significant

Higher albuminuria levels were associated with calcium channel blocker and insulin use (β coefficient: 0.49 and *p* < 0.01). Hypertension and high triglyceride levels were associated with higher albuminuria levels (β coefficient: 0.33 and 0.02; *p* = 0.03 and <0.01, respectively). The use of Metformin and RAAS blocker was negatively associated with the presence of albuminuria (β coefficient: − 0.6 and −0.33, respectively; *p* < 0.01). The serum albumin level was associated with the degree of albuminuria (a higher serum albumin level was associated with a higher degree of albuminuria) (*p* < 0.01) (Table 4).

**Table 4:** Multivariable ordinal logistic regression analyses evaluating correlates of albuminuria

Variable	Subgroups	Beta coefficient	95% CI	*p* value
**Sex**	Female (Ref)			
	Male	0.05	−0.2–0.3	0.7
**Age (Y)**		0.01	−0.01–0.02	0.4
**Duration of DM (Y)**		0.01	−0.02–0.03	0.2
**Medical history**	Smoking	0.1	−0.06–0.25	0.24
	Hypertension	0.33	0.04–0.6	0.03*
	Stroke	0.1	−0.33–0.5	0.7
	Neuropathy	0.07	−0.16–0.3	0.53
	Retinopathy	0.14	−0.1–0.4	0.27
**Medications**	Insulin	0.36	0.11–0.62	<0.01*
	Metformin	−0.62	−0.88–-0.36	<0.01*
	ACEIs/ARBs	−0.33	−0.58–-0.09	0.01*
	Beta blockers	0.22	−0.03–0.47	0.08
	CCB	0.49	0.23–0.75	<0.01*
**Triglyceride’s level (mg/dl)**		0.02	0.01–0.03	<0.01*
**Albumin (g/dl)**		−0.7	−1.0–-0.4	<0.01*

*CI* confidence interval, *DM* diabetes mellitus, *ACEI* angiotensin converting enzyme inhibitor, *ARB* angiotensin receptor blocker, *CCB* calcium channel blocker

*Statically significant

**Fig. 2:**
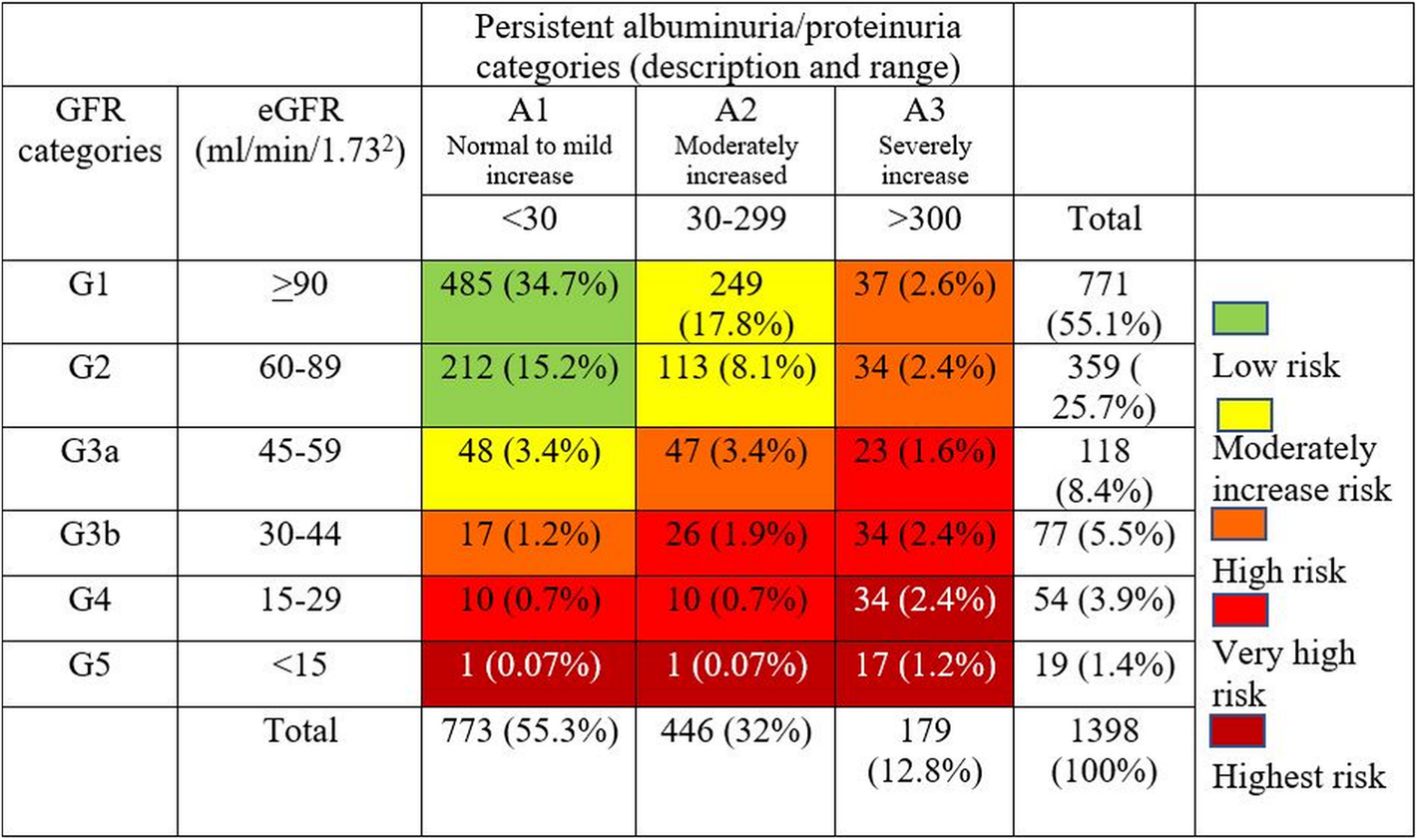
Risk categories of CKD according to 2012 KDIGO (Kidney Disease: Improving Global Outcomes) classification in subgroup of 1398 patients. Patients were categorized into each risk category based on eGFR value and severity of albuminuria

According to the Kidney Disease: Improving Global Outcomes guidelines, we categorized kidney function into CKD-G and CKD-A stages based on the eGFR and ACR [9]. We found that 29.3% of the study population had a moderate risk for all major outcomes [9] (Fig. 2) and that 5.4% of our patients (76 patients) had non-albuminuria DKD.

## Discussion

We found that approximately half of the diabetic patients visiting the endocrinology clinic had DKD. The prevalence of DKD was related to age, ranging from 46% in patients aged <60 to 52.8% in those aged >60 years. The following factors were significantly correlated with DKD: older age (>60 years), diabetic microvascular and macrovascular complications, and dyslipidemia. Metformin and RAAS blockers were associated with a lower degree of albuminuria and less advanced CKD.

Our results were in line with the reported prevalence of DKD in the Middle East (33–54.1%) [10, 11]. These studies relied on albuminuria to define DKD and this potentially underestimated the prevalence of DKD in some reports [12–14]. The reported overall prevalence in our study was also in line with that reported in the US (54%) [15]. Nevertheless, the prevalence of DKD in younger patients in the US were found to be approximately 25%, as compared with 46% in our population [15]. The higher prevalence of DKD in younger patients is alarming and might result in a public health crisis in the coming years if active intervention is not performed.

The prevalence of DKD increases with age and is more evident by a low eGFR than by albuminuria. This can be explained by the physiological decline in renal function with age, which is associated with a gradual decline in eGFR [16]. This observation was confirmed in our study, wherein the impairment of eGFR with age was more marked than the occurrence of albuminuria.

Our results showed that patients who reported using metformin or RAAS blocker had a lower risk of developing DKD and proteinuria. Metformin is the preferred treatment option for DM type 2 and we found that metformin was negatively associated with DKD, albuminuria, and advanced CKD, consistent with most of the observational studies reporting that metformin is associated with reduced progression to ESKD in type 2 diabetes patients with DKD. However, the reason for these observations remains unknown [17]. In most animal model studies, metformin has shown prominent inhibitory effects on tubulointerstitial fibrosis in both diabetic and non-diabetic models [18]. Further, the long-term benefits of metformin regarding ESKD and cardiovascular disease in patients with moderate CKD have been demonstrated. To achieve the maximal renoprotective benefits of metformin, it may be necessary to use metformin in combination with SGLT2 (Sodium-Glucose co-transporter-2) inhibitors or incretin-based therapies at an early stage of DKD [19]. Unfortunately, metformin should be discontinued in cases of advanced CKD despite this favorable early effect.

Clinical trials have also demonstrated the beneficial effects of RAAS blockers in delaying the progression of DKD. Two large randomized long-term trials, the Angiotensin Antagonist Losartan study [20] and the Irbesartan Diabetic Nephropathy Trial [21] indicated that ARBs are effective in slowing the progression of diabetic nephropathy. These studies have shown that nephroprotection with ACE inhibitors and ARBs was greater than what might be expected due to a reduction in blood pressure. Our study found that RAAS blockers were negatively associated with a lower degree of albuminuria and advanced CKD stage. Many studies have shown that ACEIs and ARBs have beneficial renoprotective effects concerning the progression of DKD in hypertensive diabetic patients and can delay or prevent the development of diabetic nephropathy independently of the beneficial blood pressure-lowering effect in patients with DM type 2 and microalbuminuria [22, 23]. Subsequently, the National Kidney Foundation has recommended using that either ARBs or ACEIs in patients with diabetes regardless of the presence of hypertension.

Albuminuria is considered the first clinical symptom of DKD and is traditionally used as a screening test for DKD. However, mounting evidence suggests that the traditional concept of the natural history of DKD has changed, and a considerable proportion of adult diabetics are normoalbuminuric despite having a low eGFR [24]. The prevalence of normoalbuminuric DKD varies from 14.29 to 56.6% among diabetic patients of different ethnicities [25, 26]. These findings have led the American Diabetes Association to recommend screening DKD based on albuminuria and eGFR to avoid missing cases. In our population, only 5.4% of patients had an eGFR of <60 mL/min/1.73 m^2^ without evidence of albuminuria.

The association between hypertension and albuminuria is well established but the mechanism is still controversial. It is thought to be an endothelial dysfunction, which is strongly associated with increased cardiovascular risk, and causes renal manifestation. Hypertension is reported in approximately 70–80% of DM type 2 at the time of diagnosis and known to exacerbate diabetic nephropathy [27]. Our study also showed that hypertension was associated with higher degree of albuminuria and DKD in patients with DM type 2.

The association between dyslipidemia and the development of DKD was investigated in several studies, and many epidemiological studies demonstrated an association between diabetic dyslipidemia and DKD [28]. Post-hoc analysis of large interventional studies of high-risk patients with diabetes [29, 30] revealed that high triglyceride and low HDL concentrations were associated with DKD. The ADVANCE study demonstrated that lower baseline HDL levels were a significant and independent predictor of DKD. In contrast, no association was found with the risk of diabetic retinopathy, suggesting that differences may exist in the pathophysiology of these microvascular complications [29]. The FELD study showed that the hypotriglyceridemic drug fenofibrate slowed the decline of renal function and reduced the degree of albuminuria in patients with DM type 2 [30]. Our study also showed that low HDL cholesterol levels and high triglyceride levels were associated with DKD.

There were several limitations to this study. First, the retrospective and the cross-sectional study design introduced recall bias and precluded temporal evaluation of risk factors and outcomes of interest. Therefore, a cause-effect relationship cannot be concluded. However, the data obtained by personal interviews and referring to medical charts minimized the recall bias and improved the data quality.

Second, our study was performed in a tertiary medical center and might not reflect the status-quo of diabetes in Jordan. Nevertheless, this tertiary center provides service to a wide range of patients with different socio-economic backgrounds all over Jordan. Additionally, given the Jordanian population’s unique composition, our findings can be generalized to the middle East. Third, this study had a small sample size for a common disease and did not examine the onset and the course of DKD in the population as the baseline readings of albuminuria and renal function at the time of diagnosis were not available.

## Conclusion

We found that more than half of the patients with DM type 2 had DKD with approximately one-third were at moderate risk of major adverse events. DKD was correlated to older age, longer duration of diabetes, the presence of diabetic complications, and dyslipidemia. In addition, patients who were using metformin or RAAS blocker had a lower risk of DKD and proteinuria. Albuminuria adds valuable information regarding the prevalence, epidemiological characteristics, and risk factors of DKD. Prospective studies with long follow-up duration are needed to understand the natural course of DKD and future research should also focus on the effect of raising awareness about DKD in patients with DM type 2 to aid in implementing a more targeted community-based intervention.

## List of Abbreviations

DKD: Diabetic kidney disease
DM: Diabetes mellitus
CKD: Chronic kidney disease
ESKD: End-stage kidney disease
HbA1c: Hemoglobin A1c
eGFR: The estimated glomerular filtration rate
ACR: Urinary albumin to creatinine ratio
PCR: The protein to creatinine ratio
RAAS: Renin-angiotensin system
BMI: Blockers, body mass index
